# Identification of the Lipodepsipeptide MDN-0066, a Novel Inhibitor of VHL/HIF Pathway Produced by a New *Pseudomonas* Species

**DOI:** 10.1371/journal.pone.0125221

**Published:** 2015-05-27

**Authors:** Bastien Cautain, Nuria de Pedro, Christian Schulz, Javier Pascual, Thiciana da S. Sousa, Jesús Martin, Ignacio Pérez-Victoria, Francisco Asensio, Ignacio González, Gerald F. Bills, Fernando Reyes, Olga Genilloud, Francisca Vicente

**Affiliations:** Fundación MEDINA, Parque Tecnológico de Ciencias de la Salud, Avda. del Conocimiento 3, 18016 Granada, Spain; UCSF / VA Medical Center, UNITED STATES

## Abstract

Throughout recent history, metabolites of microbial origin have had an extraordinary impact on the welfare of humanity. In fact, natural products have largely been –and still are– considered an exceedingly valuable platform for the discovery of new drugs against diverse pathologies. Such value is partly due to their higher complexity and chemical diversity as compared to those of synthetic and combinatorial compounds. Mutations in the Von Hippel-Lindau (*vhl*) gene are responsible for VHL disease, congenital polycythemia, and are found in many sporadic tumor types. The primary cause of morbidity and mortality for these patients arises from progression of Renal Cell Carcinoma (RCC) or end-stage renal disease. Inactivation of the Von Hippel-Lindau (*vhl*) tumor suppressor gene arises in the majority of Renal Cell Carcinoma (RCC) as well as in other types of cancer and is associated with a high degree of vascularization and poor prognosis. Loss of pVHL function thus represents a pathognomonic molecular defect for therapeutic exploitation. In this study, renal carcinoma cell lines with naturally occurring *vhl* mutations (RCC4 VA) and their genetically matched wild-type *vhl* (RCC4 VHL) counterparts were seeded onto 96-well plates and treated with a collection of 1,040 organic extracts obtained from 130 bacterial strains belonging to at least 25 genera of the phyla *Actinobacteria*, *Firmicutes*, *Proteobacteria* and *Bacteroidetes*. This strategy allowed us to identify several extracts obtained from bacterial strain F-278,770^T^, the type strain of the recently proposed new species *Pseudomonas granadensis*, showing biological activities not associated with previously known bioactive metabolites. The fractionation and structural elucidation of one of these extracts led to the discovery of a new lipodepsipeptide (MDN-0066) with specific toxicity in pVHL deficient cells that is not detectable in cells with pVHL expression rescue. This specific toxicity is associated with apoptosis induction in VHL deficient cell line as demonstrated with PARP activation and Annexin V staining. Our study demonstrated the feasibility of selectively targeting the loss of the *vhl* tumor suppressor gene for potential clinical benefit. Our results may have great impact on the development of new targeted therapies from natural products for the treatment of cancer and other genetic diseases.

## Introduction

One-third of top-selling drugs are derived from natural products. Antibacterial and antifungal antibiotics, immunosuppressors, antitumoral and hypolipidemic agents, derived from compounds of microbial origin, are increasingly used in clinical practice [1] outstanding diversity of chemical structures that microorganisms are able to produce derive from a relatively limited number of basic biosynthetic pathways (mainly polyketides, non-ribosomal peptides, terpenoids, and their combinations), that have greatly diversified through evolution. In view of such notable structural diversity, it is not surprising that we keep on finding microbial metabolites with biological activity of interest for a wide range of therapeutic fields [2,3].

Antitumoral chemotherapeutic agents of microbial origin are among the most extensively used in the clinic. Traditionally, these comprise cytotoxic compounds, mostly produced by *Streptomyces* strains, which act on DNA either as alkylating or intercalating agents that cause strand breakage, or by interfering with the replication process. The most important compounds with clinical application are the anthracyclines daunomycin and their derivative doxorubicin, used in the treatment of leukemias, non Hodking lymphomas and breast cancer; aclarubicin (or aclacinomycin A), which represents a novel type of topoisomerase I and II inhibitors, and presents activity against leukemias and solid tumors; glycopeptidic bleomycins A2 and B2, used for the treatment of squamous cell carcinomas and lymphomas; peptides like actinomycin D; quinones like mitomycin C; and mithramycin, an extremely cytotoxic compound whose clinical use has been restricted to the treatment of certain tumors, such as testicle carcinome due to its severe side effects [1]. The identification of novel chemical structures with biological activity is still an urgent task in many therapeutic areas, and innovative strategies are constantly under development. In the case of natural products such strategies aim at expanding the chemical diversity of natural compounds and range from the exploration of non-conventional sources of natural compounds, such as algae and insects, to the development of alternative approaches for the generation of libraries of compounds by means of total synthesis inspired by natural products scaffolds, semi-synthesis or combinatorial libraries obtained by heterologous expression of biosynthetic pathways. These efforts, together with the identification of novel targets and the development of innovative assays which can be easily adapted to HTS technology, constitute the key to the current drug discovery programmes.

The molecular basis of human cancers has been greatly illuminated by the identification of familial cancer syndrome genes. Despite their rarity, the genes that are linked to hereditary cancer syndromes are almost invariably important in fundamental cellular processes such as cell growth, division and apoptosis that are commonly disrupted in human cancers. Consequently, investigations of the specific mutations responsible for these syndromes combined with the cellular signaling networks disrupted by the mutant proteins have provided unprecedented insights into the molecular origins and pathogenesis of inherited and sporadic forms of cancer and, perhaps as important, offering new avenues for understanding basic biological processes. Studies of the Von Hippel Lindau (VHL) cancer syndrome illustrate these principles very well. Two eye doctors—von Hippel in Germany and Lindau in Sweden—were the first to publish descriptions of tumors in patients' eyes and brains, hallmarks of von Hippel-Lindau syndrome [4]. This syndrome is caused by inactivating germline mutations in the *vhl* gene and associated with an increased risk of a variety of tumors including clear-cell renal carcinoma in an allele-specific manner. The protein encoded by this gene is a component of the protein complex that includes elongin B, elongin C, and cullin-2, and possesses ubiquitin ligase E3 activity. This protein is involved in the ubiquitination and degradation of hypoxia-inducible-factor (HIF), which is a transcription factor that plays a central role in the regulation of gene expression by oxygen [5,6,7]. Clear cell renal cell carcinoma (CC-RCC) is a prominent feature in hereditary VHL disease, suggesting an etiologic role of the *vhl* gene in sporadic CC-RCC. Gnarra et al. [8] found that *vhl* is mutated in 57% of CC-RCC. An additional 10% to 20% of cases are due to inactivation of the *vhl* gene through hypermethylation. Thus, loss of pVHL function occurs in a significant fraction of sporadic CC-RCC, approaching 70% to 80% of all cases [9].

It is in this context that the development of bioassays based on cells expressing or not pVHL has a series of remarkable advantages, since it is suitable for high-density formats, detection methods which are easy to perform and interpret (e. g. measurements of cell growth by spectrometry), promptness in the acquisition of results, etc. The *vhl* gene is ubiquitously expressed in normal tissues throughout the body; thus, loss of *vhl* expression is unique to tumor pathology. The high frequency of pVHL inactivation in RCC makes it nearly universal to the disease state and tumor suppression following reintroduction of *vhl* into RCC cells underscores the crucial role of pVHL in malignancy. A molecular defect that is crucial to the malignant phenotype, unique to diseased tissue, and nearly universal to the disease state serves as an ideal target for therapeutic intervention. Pharmacologic agents that are toxic in the context of pVHL disruption should then have minimal effect on normal tissue.

In this study, RCC cells deficient in *vhl* transformed with vector alone or *vhl* expressing vector were used as a platform for studying an extensive collection of microbial extracts obtained from unicellular bacteria in search for new anticancer agents. This bioassay was directed to the identification of novel anticancer compounds and is based on specific toxicity in *vhl* deficient cells that is not detectable in cells with *vhl* expression rescue [10]. Our group was the first to use this methodology for screening a collection of bacterial natural products extracts, including new bacterial taxa, belonging to the phyla *Actinobacteria*, *Firmicutes*, *Proteobacteria* and *Bacteroidetes*.

MDN-0066, the bioactive compound identified could provide very useful tools for in depth investigation of the function of their targets. Furthermore, the results obtained in this work could be of significance for the future development of novel therapies.

## Materials and Methods

### Cell culture and stable transfections

Human Renal Cell Carcinoma Cell line RCC4 stably transfected with an empty vector, pcDNA3 (ECACC N-03112702; called RCC4-VA) conferring neomycin resistance, or with pcDNA3-VHL (ECACC N-0312703; called RCC4-VHL) conferring neomycin resistance and encoding the VHL tumor suppressor gene product pVHL were used in this study. The original renal carcinoma cell line RCC4 is VHL-deficient. The RCC4 plus vector alone cell line serve as a negative control cell line to study the effect of pVHL expression from pcDNA3-VHL. The human renal carcinoma cell lines RCC4-VA and RCC4-VHL were cultured at a density of 1.0 × 10^6^ cells/ flask in BD Falcon Tissue Culture Flasks in Dulbecco’s modified Eagle’s medium, supplemented with 10% fetal bovine serum (Origin: Australia GIBCO), 0.01% L-Glutamine 200 mM, 100 × (GIBCO), 0.01% penicillin streptomycin (GIBCO), 0.001% geneticin, G418 0.5 mg/ml (GIBCO). Cell cultures were maintained in a humidified incubator at 37°C with 5% CO_2_ and passaged when confluent using TrypLE Express 1 × (GIBCO). Cells were counted by means of trypan blue and hemocytometer.

Human breast adenocarcinoma-derived MCF7 cell line, Human hepatocellular carcinoma cell lines HepG2, human osteosarcoma cell line U2OS and human pancreatic cancer cell line MiaPaCa-2, were purchased from ATCC (American Type Culture Collection, Manassas, VA, LGC Standards) and were maintained in Dulbecco's Modified Eagle Medium (DMEM) or Roswell Park Memorial Institute medium (RPMI) (Gibco, Life Technologies, Milan, Italy) that were supplemented with 10% Fetal Bovine Serum (FBS), 100 units/ml penicillin, 100 μg/ml streptomycin, and 2 mM L-Glutamine. Cells were grown in humidified atmosphere at 37°C with 5% CO_2_ in a cell culture incubator.

### Validation and Automation of the RCC4-VA/VHL Assay

**Fig 1:**
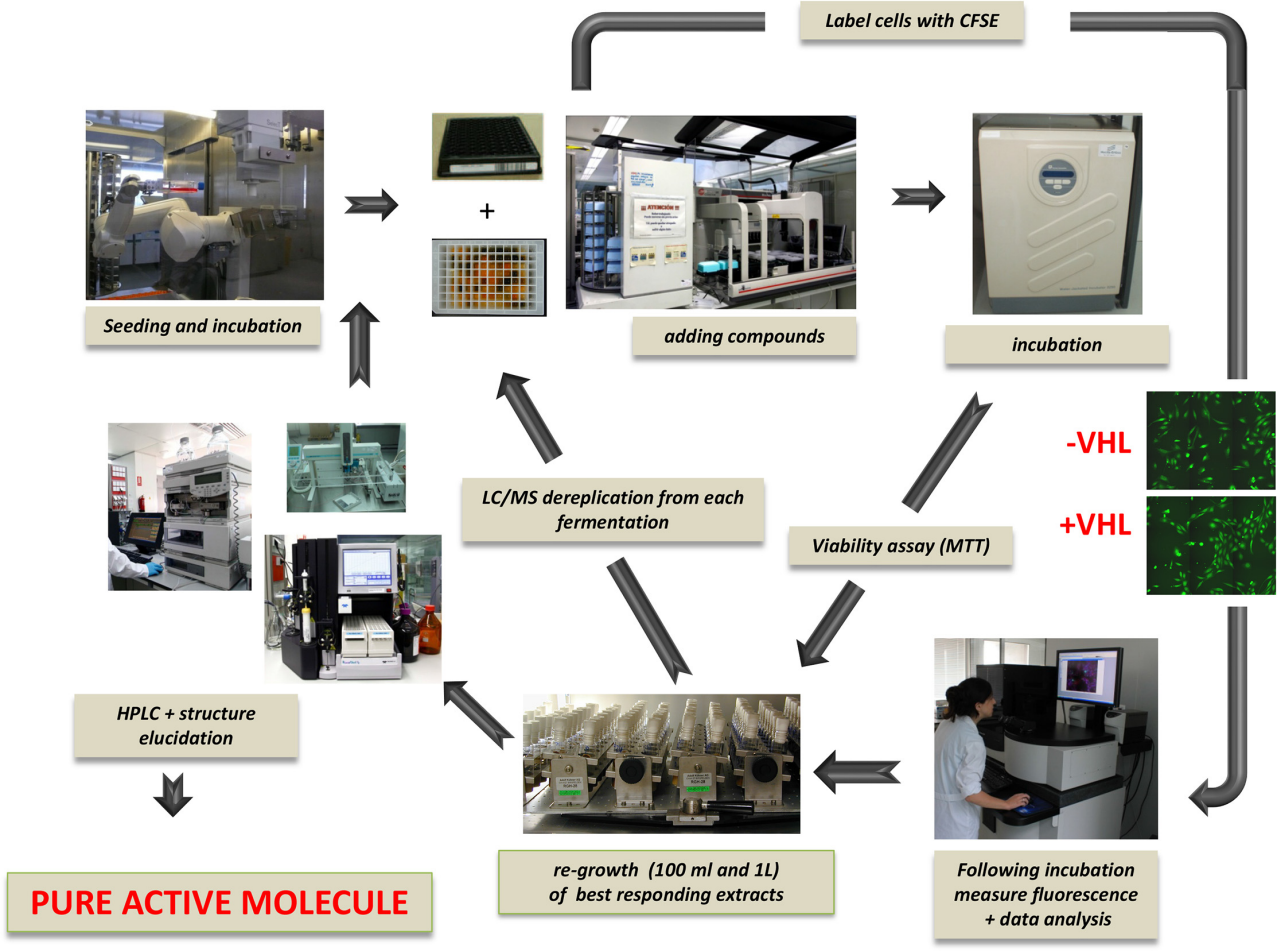
Automated WorkFlow. The automated workflow for the RCC4VA/VHL assay treated with natural extracts allows for the standardization of the process. Cells were seeded automatically and incubated overnight. The compounds or natural extracts from bacteria or fungi were transferred from mother plates to assay plates using a robotic workstation. After 24 hours of incubation at 37°C in medium containing the extracts or pure compounds cells were washed, fixed, and stained in a fully automated manner using a robotic workstation. For automated microscopy we used BD Pathway 855 High Content Bioimager.

The automated screening process was formatted in 96-well plates (Greiner Bio-One, Frickenhausen, Germany), and workflow was automated (Fig 1). Cells were seeded at a density of 20,000 cells per well using a multidrop dispenser (MTX Lab Systems, Inc. Vienna, VA, USA). All liquid handling for compound treatment, washing, fixing, and staining steps were performed by a robotic workstation (cell culturing robot, Select T; TAP Biosystems, Royston, UK). All experiments were performed in triplicate.

### Cell Viability and XTT assays

MTT (3-(4,5-Dimethylthiazol-2-yl)-2,5-diphenyltetrazolium bromide) is a colorimetric assay for measuring the activity of cellular enzymes that reduce the tetrazolium dye, MTT, to its insoluble formazan, giving a purple color. This assay measures mitochondrial metabolic activity via NAD(P)H-dependent cellular oxidoreductase enzymes and may, under defined conditions, reflect the number of viable cells [11].

Cells were seeded at a concentration of 1× 10^4^ cells/well in 200 μl culture medium and incubated at 37°C in 5% CO_2_ using 96 wells microplates (BD Falcon). After 24 hours, the medium was replaced with a final volume of 195 μl and 5 μl of extracts and controls were added to the plates. 8 mM methyl methanesulfonate (MMS) acts as a positive control and 0.5% DMSO as a negative control. On the last column there are four points of rotenone and doxorubicin with an initial concentration of 10 mM and dilution ½. When extracts and controls were added, plates were incubated at 37°C in 5% CO_2_ incubator for 24 hours. After this time, an MTT solution was prepared at 5 mg/ml in PBS 1x and then diluted at 0.5 mg/ml in MEM without phenol red. The sample solution in wells was flicked off and 100μl of MTT dye was added to each well. The plates were gently shaken and incubated for 3 hours at 37°C in 5% CO_2_ incubator. The supernatant was removed and 100 μl of DMSO 100% was added. The plates were gently shaken to solubilize the formed formazan. The absorbance was measured using a multireader named Victor2 (Wallac) at a wavelength of 570 nm.

### Microbial extracts collection

A collection of 1,040 acetone extracts was generated after cultivating 117 unicellular bacteria in eight production media. Bacterial strains were isolated by using improved culture conditions such as long-term incubation times, culture media with unusual nutrients at oligotrophic concentrations, use of gellan gum as solidifying agent and diluted bacterial inocula. The molecular identification of isolates based on the 16S rRNA gene sequences of the isolates was done through the on-line tool EzTaxon (http://eztaxon-e.ezbiocloud.net/). Isolates were classified into at least 25 genera belonging to phyla *Actinobacteria*, *Firmicutes*, *Proteobacteria* and *Bacteroidetes* (data not shown).

### Bioassay guided fractionation of extracts

Active extracts were fractionated on a Gilson Preparative HPLC system using a Zorbax RX-C8 column (9.4 × 250 mm, 5 μm), a gradient H_2_O/CH_3_CN from 5 to 100% CH_3_CN in 35 min + 5 min at 100% CH_3_CN, a flow rate of 3.6 mL/min, and UV detection at 210 and 280 nm. A total of 80 fractions were collected every 30 seconds from each chromatographic run, dried under vacuum, and tested for their RCC4-VA RCC4-VHL activity in the above mentioned conditions. Active fractions from each extract were analyzed by LC/HRMS and NMR in order to establish the identity and/or novelty of the bioactive components.

### Scale-up fermentation of bacterial strain F-278,770^T^

The first seed culture of the strain was prepared by inoculating 10 mL of seed medium R2A (BD Difco) in three 50 mL tubes with frozen glycerol inoculum stock of the bacterial strain and incubating the tubes at 18°C with shaking at 220 rpm for 48 hours. A second seed culture was prepared by inoculating 50 mL of seed medium in three 250 mL flasks with 5 mL of the first seed. A 10% aliquot of the second seed culture was transferred to each of the twenty one 500 mL flasks containing 150 mL of the production medium (Naganuma medium) consisting of glucose (0.1 g/L), potato starch (0.3 g/L), peptone (0.05 g/L), soybean flour (0.1 g/L), yeast extract (0.05 g/L) and adjusted to 7.0 before the addition of CaCO_3_ 0.02 (g/L). The flasks were incubated at 18°C for 72 hours in a rotary shaker at 220 rpm and 70% humidity before harvesting.

### Bioassay-guided purification of MDN-0066

A 3L fermentation was acidified to pH 3 with HCl 6N. The acidified fermentation was extracted with methylethylketone (MEK, 3L) under shaking at 220 rpm for 1 h. The aqueous phase was then separated by centrifugation and discarded, and the organic phase (ca. 3L) was concentrated to dryness on a rotary evaporator. The dried extract was fractionated by reversed-phase preparative HPLC (Agilent Zorbax SB-C8, 21.2 x 250 mm, 7 μm; 20 mL/min, UV detection at 210 nm, gradient H_2_O + 0.1%TFA:CH_3_CN + 0.1%TFA from 5% to 100% organic in 40 minutes) yielding a fraction eluting at 29.5 min that was further purified by semipreparative HPLC (Agilent Zorbax RX-C8, 9.4 x 250 mm, 5μm; 3.6 mL/min, UV detection at 210 nm, gradient H_2_O + 0.1%TFA:CH_3_CN + 0.1%TFA from 75% to 80% acetonitrile in 40 minutes) to yield 52.8 mg of MDN-0066 as a white amorphous solid. All fractionation steps were guided by testing of inhibitory activity in the RCC4-VA/VHL assays.

### Determination of IC_50_ values in RCC4 assays

Determination of IC_50_ values were carried out in RCC4 assays. Cells were cultured as described above and exposed for 24 hours to equal volumes of test compounds (2 μL). IC_50_ values were calculated as being the inhibitor concentration that decreases 50% of the cell viability using Genedata Screener software (Genedata AG, Switzerland). Cells were treated with eight 2-fold serial dilutions of each compound spanning concentrations from 200 μM to 1.5 μM in 1% DMSO final. Compound activity was normalized to the in-plate negative and positive controls using DMSO and LMB, respectively. We chose 4 mM MMS as maximal inhibition and 1% DMSO as minimal inhibition of RCC4-VA/VHL assays.

### Immunoblotting

Cells were seeded at a density of 5.0 × 10^5^ cells/well into 6 wells microplates (BD Biosciences, San Jose, CA) and incubated at 37°C and 5% CO_2_. After 24 hours, cells were treated with 25 mM MDN-0066 and with DMSO as a negative control and incubated at 37°C for 24 or 48 hours. Cells were lysed in cell lysis buffer (Cell Signaling Technology, Danvers, MA). Twenty to fifty micrograms of protein (as determined by Pierce BCA Protein Assay Kit, Thermo Scientific) were resolved on 12% SDS-polyacrylamide gels and then transferred onto to a pre-wetted in methanol PVDF membrane that was blocked using SEA BLOCK Blocking Buffer (Thermo Scientific). Total HIF-1α, MCT4, C-Myc, Beclin, LC3b, LDHA, cleaved PARP (Cell Signaling Technology, Danvers, MA) and PKM2 (ProteinTech, Chicago, Illinois) were used as primary antibodies. β-actin (Sigma-Aldrich, St Louis, MO) was used as housekeeping protein for normalization. Anti-mouse and anti-rabbit DyLight 700 and 800 conjugated (Cell Signaling Technology, Danvers, MA) were used as secondary antibodies. Membrane was washed and read in Odyssey Infrared Imaging System (LI-COR Bioscience, Lincoln, Nebraska USA).

### Flow cytometry

Cellular DNA content was determined by means of flow cytometric analysis, using a DNA- specific fluorescent dye, propidium iodide (PI). The cells were plated in a six-well plate and cultured for 48 h. The cultured cells were treated with DMSO or MDN-0066 compound 48 h, cells were washed with phosphate-buffered saline (PBS) and trypsinized. After centrifugation, 1 mL of -20°C 70% EtOH was added dropwise to the cell pellet. The cells were stored at -20°C until the day of DNA staining. On the day of DNA staining, the samples were washed with PBS and suspended in 1 mL of DNA staining buffer containing PI, and ribonuclease-A for incubation for 1 h at room temperature. Samples were analyzed by BD Accuri C6 cytometer. For each experiment 20,000 events were counted, and the percentages of the cells in the different cell-cycle phases (subG1, G1, S and G2/M) were determined.

Cell apoptosis was also assessed by flow cytometry. After treatment, approximately 1× 10^6^ cells were harvested, washed twice with pre-chilled PBS, resuspended in 300 mL binding buffer and then incubated with annexin V / propidium iodide (PI) (BD PharMingen, San Diego, CA, USA) for 20 min in the dark. The samples were analyzed using BD Accuri C6 cytometer (BD Biosciences, USA) within 1 h.

## Results

### Screening campaigns

Microbial natural products have been one of the major sources of novel drugs, and the untapped chemical diversity in these extracts favors the identification of novel molecules with potential therapeutic applications which are still waiting to be discovered from these natural sources. However, the discovery of novel RCC4-VA/VHL inhibitors from natural products has not been extensively pursued so far. Once the assay conditions were optimized, 1,040 different microbial extracts were analyzed in triplicate for their capacity to induce cell death in RCC4-VA but not in RCC4-VHL cells as described in the Materials and Methods. The standard deviations of the triplicates ranged from ± 3% to ± 5%. A relatively high number of natural extracts (0.9%) displayed inhibitor activity in RCC4- VA but not in RCC4-VHL in the primary screening.

Activity and reproducibility of the assay was confirmed in test using a second aliquot of the same extracts. In order to avoid re-isolation of previously known natural products which often occur in the course of bioassay-guided fractionation [12], active extracts or subsequently detected fractions were subjected to chemical dereplication (Fig 1). The detection of known secondary metabolites, allowed us to focus on the most promising candidates. Following this multi-step process, we selected one active extract produced by strain F-278,770^T^ to carry on with the scale-up fermentation, bioassay-guided isolation and structural elucidation processes. Several extracts obtained from strain F-278,770^T^ cultivated in Naganuma medium at 18°C displayed a specific activity against RCC4-VA/VHL. Strain F-278,770^T^ was isolated from a soil sample collected from the Tejeda, Almijara and Alhama Natural Park, Granada (Spain) in November of 2011. The strain was isolated from gellan gum-solidified VL70 isolation medium containing D-xylose, 0.05% (w/v) as carbon source [13] after an incubation period of two weeks at 18°C. After a polyphasic characterization including phenotypic, chemotaxonomic, genetic and phylogenetic studies, we concluded that our isolate constituted a new bacterial species belonging to the genus *Pseudomonas* for which the named *P*. *granadensis* sp. nov. has been proposed. The formal description of *P*. *granadensis* sp. nov. will be published elsewhere (Pascual et al., 2014).

### Isolation and structural elucidation of MDN-0066

**Fig 2:**
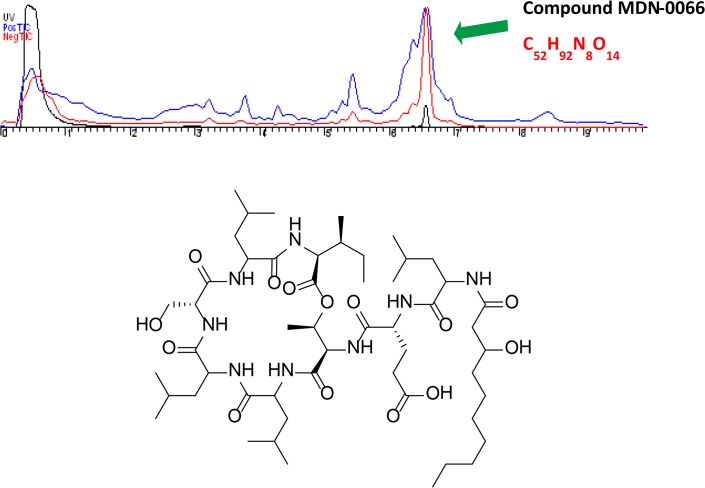
Chemical structure of MDN-0066.

An acidified fermentation broth of strain F-278,770^T^ was extracted with methylethylketone and the crude was subjected to several steps of preparative and semi-preparative reversed-phase HPLC to yield MDN-0066. A molecular formula of C_52_H_92_N_8_O_14_ was inferred from analysis of ESI-TOF MS data (Fig 2). This molecular formula together with the existence of an abundant number of signals in the amide NH and α-amino proton regions of its ^1^H NMR spectrum and carbonyl groups in the ^13^C NMR spectrum (Table 1, S1 and S2 Figs) evidenced the peptidic nature of the compound. MS/MS fragmentation of the molecule revealed the sequence Leu/Ile-Leu/Ile-Ser-Leu/Ile-Leu/Ile-Thr-Glu-C_16_H_30_NO_3_ (S3 Fig). Analysis of 2D NMR spectra, including COSY, HSQC and HMBC, confirmed the presence in the structure of Leu (4), Ile (1), Ser (1), Thr (1), Glu (1) and a lipidic residue that was identified as 3-hydroxydecanoic acid (HDA). The sequence established by MS/MS analysis was corroborated by correlations observed in the HMBC and NOESY spectra (S4 Fig). Analysis of these spectra also determined the position of the Ile residue and identified the fragment C_16_H_30_NO_3_ detected by MS/MS as Leu-HDA. Apart from the HMBC correlations observed between the β-proton of Thr and the carbonyl carbon of Ile, the existence of an ester linkage between both residues was further supported by the low field chemical shift (5.01 ppm) of the Thr oxygenated proton. Interestingly, the number of aminoacid residues and the size of the cycle place the structure of MDN-0066 within a new structural class never previously reported in *Pseudomonas* lipodepsipeptides.

**Table 1:** NMR spectra of MDN-0066, DMSO-d_6_, 24°C, (500 MHz).

Residue	C#	δ_C_, mult.	δ_H_, mult. (*J* in Hz)	Residue	C#	δ_C_, mult.	δ_H_, mult. (*J* in Hz)
HDA	1	172.1, C		Leu-2	NH		7.37, broad s
	2	43.6, CH_2_	2.25, m		*α*	52.8, CH	4.12, m
	3	68.0, CH	3.81, m		*β*	40.6, CH_2_	1.47, m
	4	36.8, CH_2_	1.34, m		*γ*	24.1, CH	1.48, m
	5	25.0, CH_2_	1.23, m; 1.35, m		*δ*	23.1^c^, CH_3_	0.84, m
	6	28.8^a^, CH_2_	1.23, m		*δ ‘*	22.1, CH_3_	0.89, m
	7	29.2^a^, CH_2_	1.23, m		CO	173.6, C	
	8	31.4, CH_2_	1.23, m				
	9	22.2, CH_2_	1.25, m	Leu-3	NH		9.02, d (6.4)
	10	14.0, CH_3_	0.84, m		*α*	52.8, CH	3.97, ddd (7.0, 7.0, 7.0)
					*β*	39.2, CH_2_	1.60, m
Leu-1	NH		8.19, d (6.9)		*γ*	24.2^b^, CH	1.67, m
	*α*	51.7, CH	4.18, m		*δ*	23.1^c^, CH_3_	0.90, m
	*β*	40.1, CH_2_	1.45, m		*δ ‘*	20.4, CH_3_	0.80, m
	*γ*	24.3^b^, CH	1.64, m		CO	172.4, C	
	*δ*	23.1^c^, CH_3_	0.88, m				
	*δ ‘*	21.4^d^, CH_3_	0.83, m	Ser	NH		7.94, d (7.7)
	CO	173.0, C			*α*	57.2, CH	4.23, m
					*β*	60.4, CH_2_	3.77, m; 3.72, dd (10.6, 4.1)
Glu	NH		8.02, d (6.9)		CO	169.3^e^	
	*α*	53.0, CH	4.11, m				
	*β*	26.3, CH_2_	1.77, m; 1.92, m	Leu-4	NH		7.53, d (9.3)
	*γ*	30.2, CH_2_	2.24, m		*α*	52.3, CH	4.29, m
	*δ*	173.9, C			*β*	42.5, CH_2_	1.40, m; 1.47, m
	CO	171.4, C			*γ*	23.7, CH	1.57, m
					*δ*	23.0^c^, CH_3_	0.83, m
Thr	NH		8.10, d (8.0)		*δ ‘*	21.3^d^, CH_3_	0.79, m
	*α*	58.4, CH	4.20, m		CO	171.4, C	
	*β*	70.6, CH	5.03 (app quin, 6.2)				
	*γ*	17.7, CH_3_	1.14, d (6.2)	Ile	NH		7.16, d (8.7)
	CO	169.3^e^, C			*α*	56.5, CH	4.31, m
					*β*	36.6, CH	1.91, m
					*γ*	24.7, CH_2_	1.11, m; 1.43, m
					*δ*	11.2, CH_3_	0.82, m
					*β*-CH_3_	15.3, CH_3_	0.84, m
					CO	169.4^e^, C	

^a, b, c, d, e^ Assignments may be interchangeable.

Attempts to establish the absolute configuration of the amino acid residues present in the structure of MDN-0066 were made using Marfey’s analysis [14]. The existence of peaks for both D and L-Leu residues in a proportion of 1:1 confirmed the presence of 2 Leu residues of each type and prevented the full assignment of the absolute configuration of the compound. D-Asp, D-*allo*-Thr, D-Ser and L-Ile were identified as components of the structure. Unfortunately, partial hydrolysis of the peptide followed by Marfey’s analysis of the fragments [15] did not allow the assignment of the full configuration of the Leu amino acid residues and it remains undetermined.

### Pure compound MDN-0066 dose-response analysis

**Fig 3:**
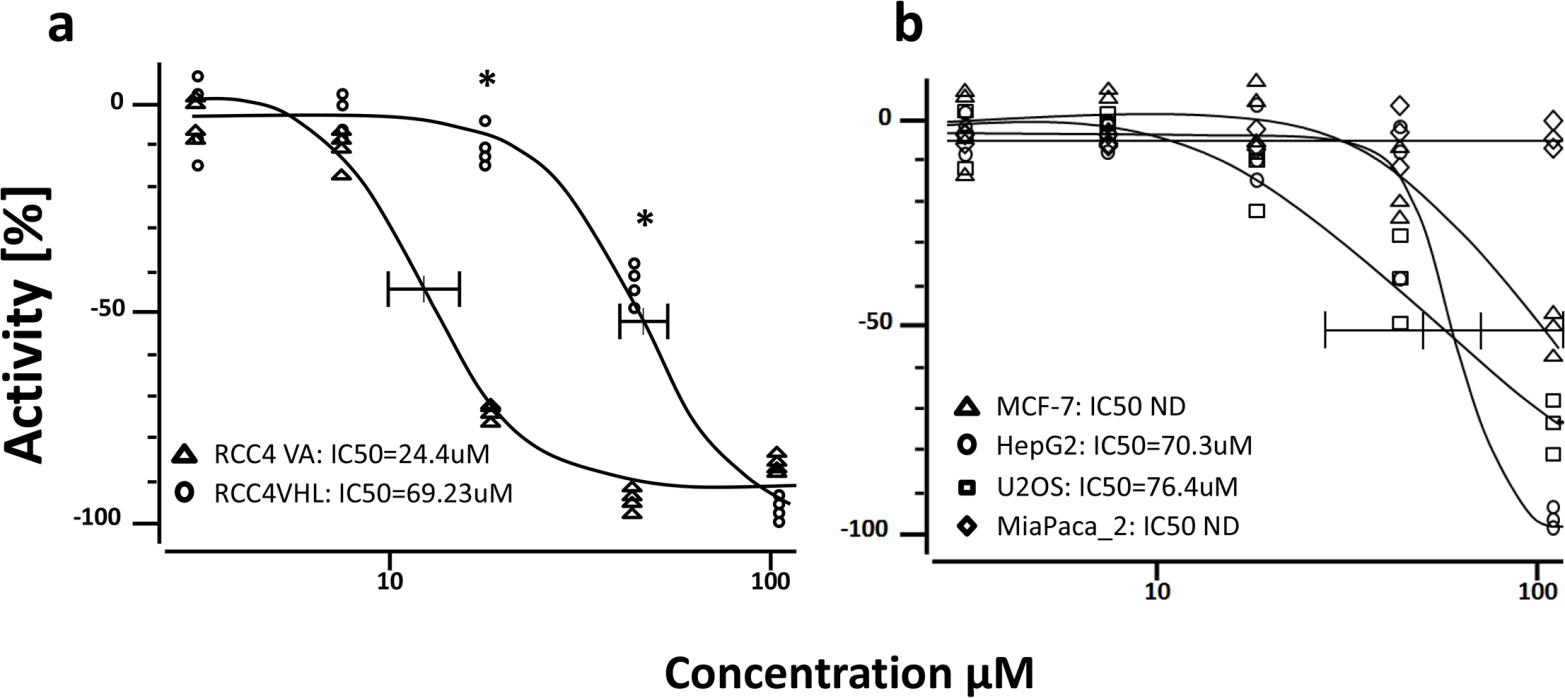
Dose–response of the active compound. Dose–response of the active compound inhibition of RCC4 VA/VHL measured in HTS format. Cells were seeded automatically at appropriate density in 96-well black-wall clear-bottom tissue culture plates and allowed to attach overnight. Cells were then treated with different concentrations of MMS (100% inhibition control) or active compound for one hour. Graphs represent the growth of the cells relative to cells treated only with carrier. Each curve is the average of triplicate samples performed independently. IC_50_ for each curve is respectively 24.4 μM and 69.23 μM for RCC4VA and RCC4VHL(*p<0.0005). Represent results of one of 3 independent experiments.

To determine the 50% inhibitory concentration (IC_50_) value of MDN-0066 in the RCC4-VA/VHL assay, cells were cultured as indicated in Materials and Methods and treated with different doses of MDN-0066 for 24 hours. Fig 3 shows that RCC4VA is much more sensitive to the presence of MDN-0066 than RCC4-VHL where vhl gene is rescued. Our experiment demonstrated that the lowest concentration of MDN-0066 that induces RCC4-VA cell death was 10 μM and a complete inhibition was observed at 50 μM while these values are respectively, 50 μM and 100 μM, for RCC4-VHL cells. IC_50_ for MDN-0066 was 24.4 μM for RCC4-VA and 69.23 μM for RCC4-VHL in this assay (Fig 3A). These values demonstrate that MDN-0066 was able to inhibit the RCC4-VA in concentrations which are not effective for RCC4-VHL. Cell viability results, as measured by MTT assay, with other cell lines (Human breast adenocarcinoma-derived MCF7 cell line, Human hepatocellular carcinoma cell lines HepG2, human osteosarcoma cell line U2OS and human pancreatic cancer cell line MiaPaCa-2 which do not present VHL mutation) indicated that MND-0066 activity is higher in RCC4 without VHL than in all other tested cell lines (Fig 3B).

### MDN-0066 affects the cell cycle of RCC4-VA cells

**Fig 4:**
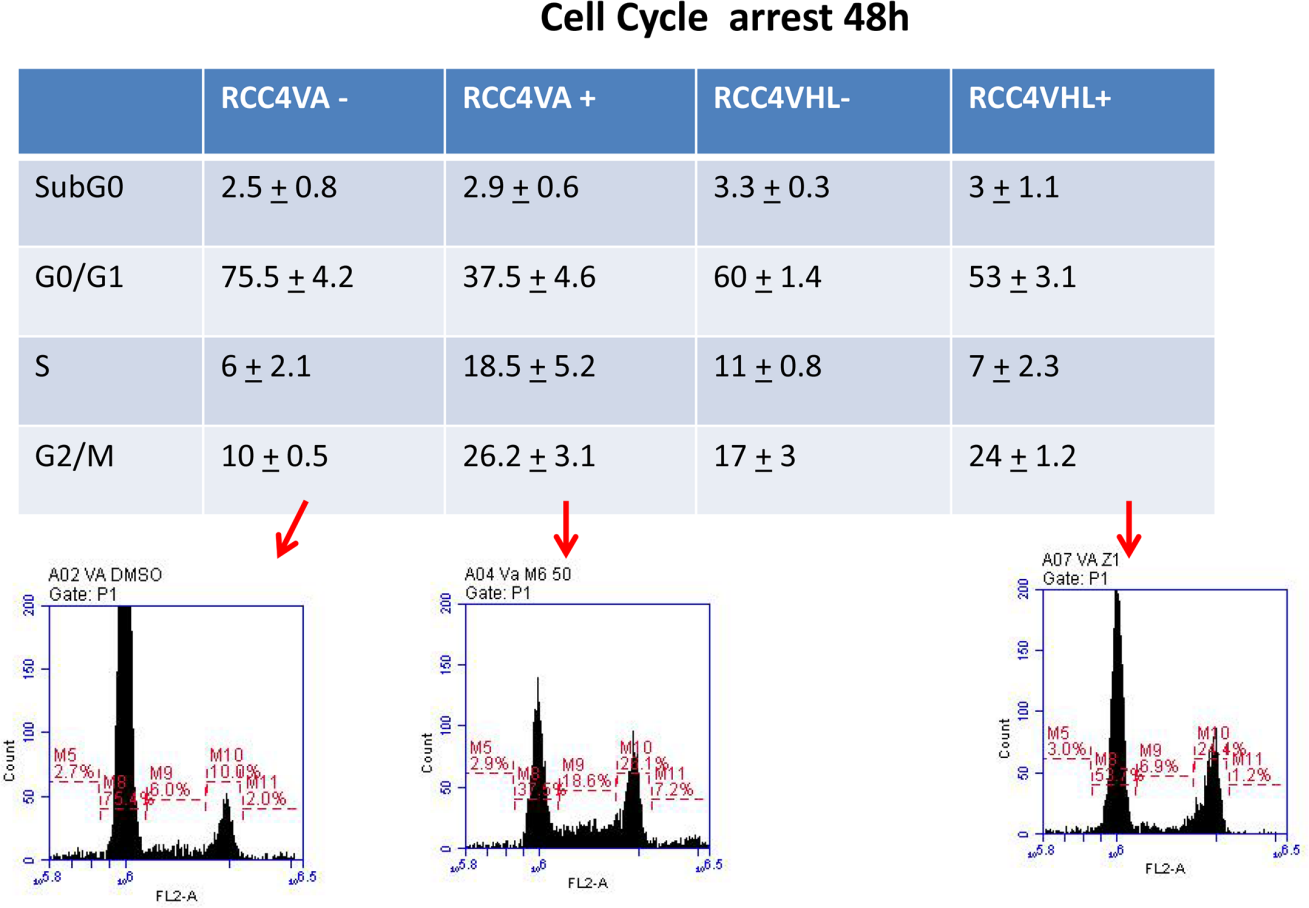
MDN-0066 affects the cell cycle of RCC4-VA/VHL cells. Cell cycle of RCC4-VA and RCC4-VHL cells after MMDN-0066 treatment. Cell cycle distribution was measured by flow cytometry using a PI Flow Kit and the percent of the cell cycle phase is shown in a table form with the SubGo, G0/G1, S and G2/M phases for the two cell lines in the absence or following 48h incubation with MDN-0066 at 24 uM. Represent results of one of 3 independent experiments.

Cell cycle checkpoints have evolved as a network of cellular signaling pathways to ensure accuracy of DNA replication and segregation of chromosomes at mitosis. These cell cycle checkpoints arrest or delay cell cycle progression either in the G1 phase before DNA replication, in S phase during DNA replication, or in G2 phase before mitosis [16,17]. Dysregulation of cell cycle checkpoints can increase susceptibility to mutations, genomic instability and tumorigenesis [18,19]. Finding cell cycle checkpoint regulators is thus of great interest in order to better manipulate the pathways for cancer therapy. Results in Fig 4 showed an S phase arrest of RCC4-VA (from 6% to 18.5%; p<0.005) cells following a 24h incubation with MDN-0066 at 24 μM while cell cycle of RCC4-VHL cells is not affected. This S phase arrest is associated with a G0/G1 decrease (75.5 ± 4.2 to 37.5% ± 4.6; p = 0.001) and a G2/M increase (10 ± 0.5 to 26.2% ± 3.1; p<0.0005). These results suggest that MDN-0066 is critical for the S phase arrest in RCC4-VA cells but can also be involved in G2/M arrest in the absence of *vhl*. Loss of HIF-1α causes an increased progression into S phase, rather than a growth arrest [20] and *vhl* is associated with cell cycle arrest upon serum withdrawal [21]. These results suggest that MDN-0066 affects the cell cycle regulation pathway of the VHL/HIF pathway.

### MDN-0066 western blot and Annexin V analysis

**Fig 5:**
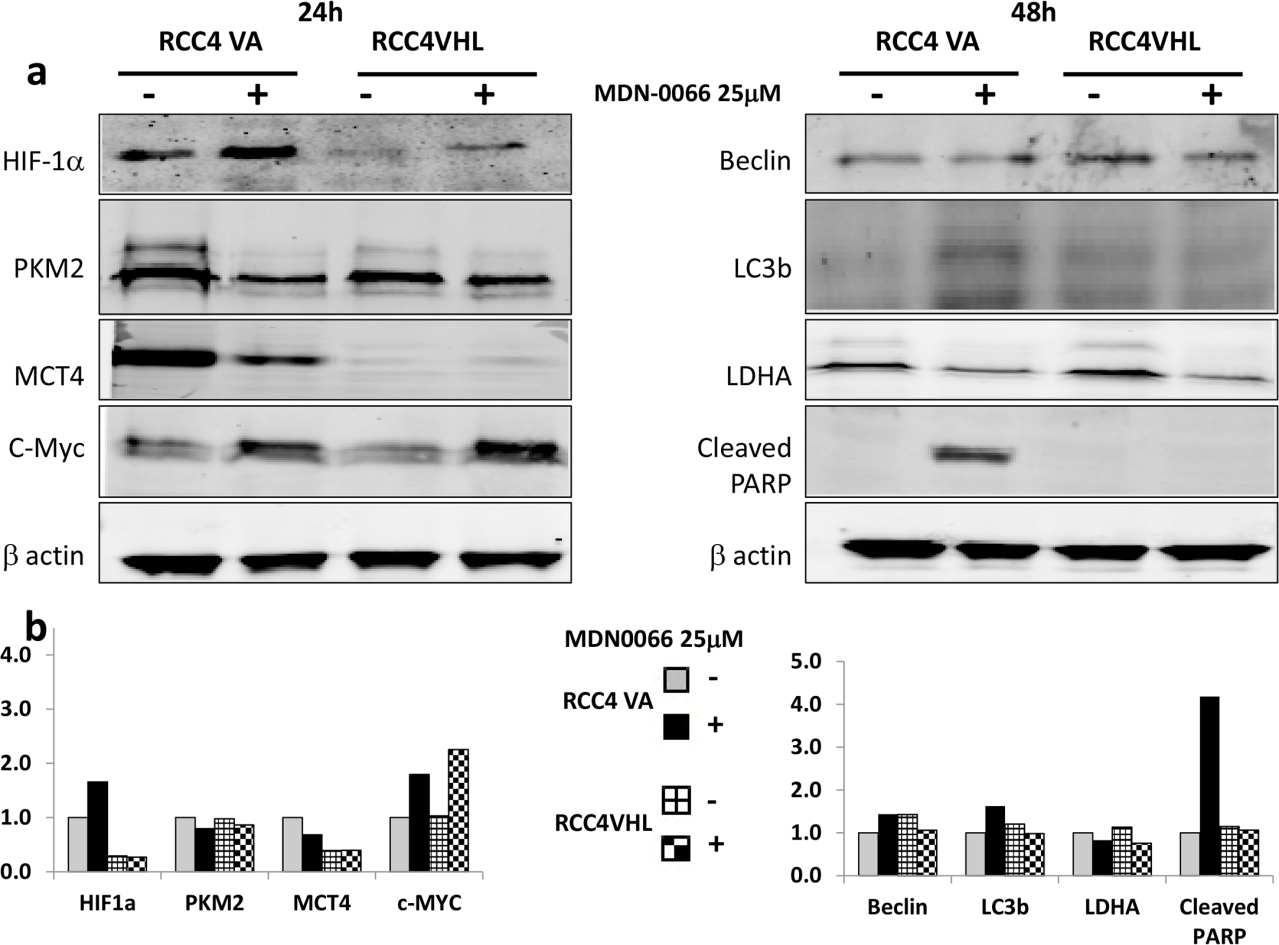
MDN-0066 western blot analysis. Cell lysates were collected from RCC4-VA and RCC4-VHL cells 24h or 48h after MDN-0066 treatment at 25uM for 24hrs or 48hrs. Blots were probed with antibodies targeting either total β-actin, HIF-1α, PKM2, MCT4, C-Myc, Beclin, LC3b, LDHA or cleaved PARP.-, no treatment, **+** MDN-0066. In Fig 5b we quantified the intensity of the western blot for each bands density using the LI-COR Aerius software.

**Fig 6:**
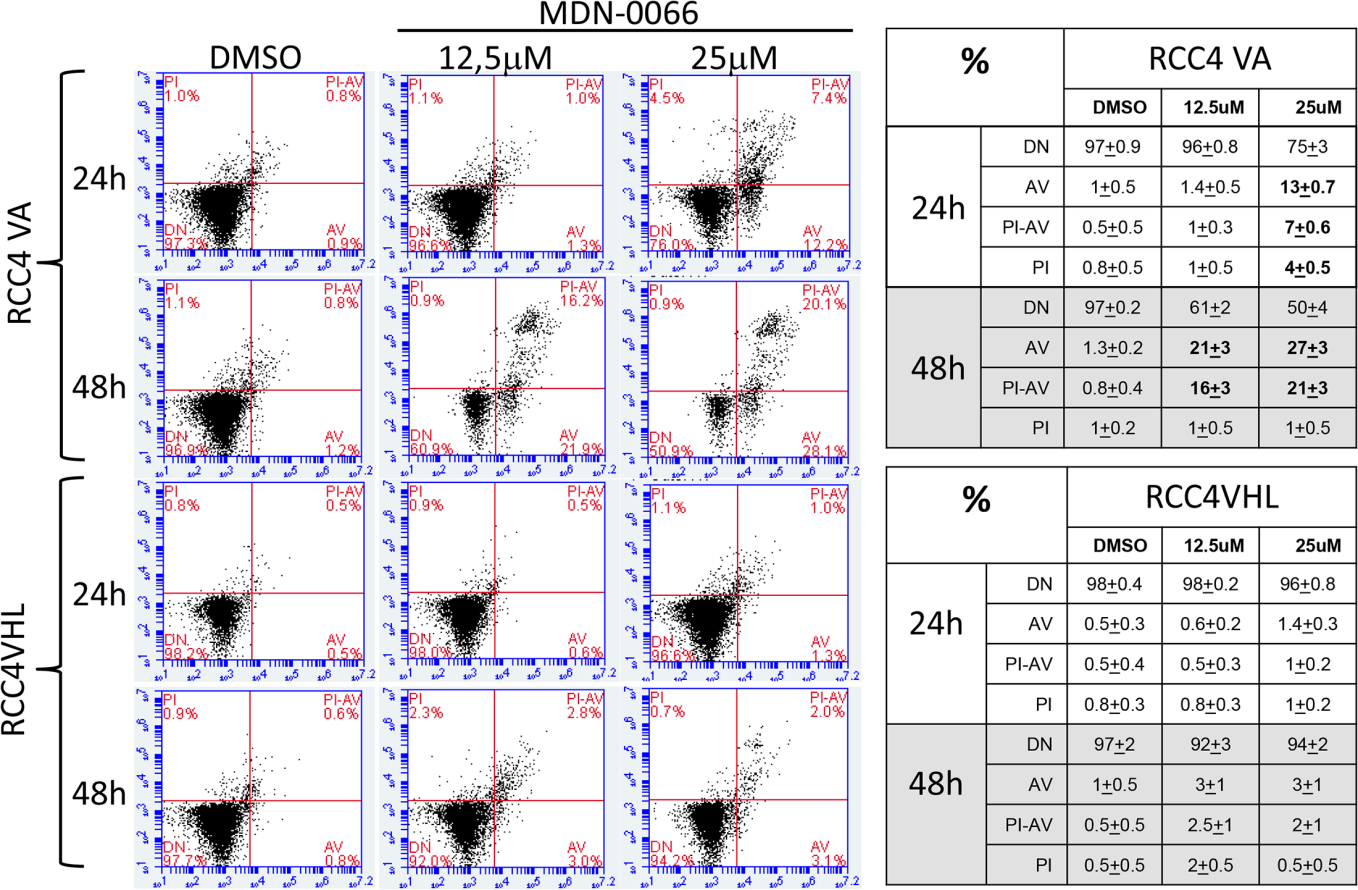
MDN-0066 cytometry analysis. Annexin-V staining of RCC4-VA and RCC4-VHL cells cultured after 24 or 48h treatment. Cells were treated with 12.5 or 25 μM MDN-0066. The analysis allows to distinguish in the diagram between living cells (lower left quadrant), early apoptotic cells (lower right quadrant), apoptotic cells (upper right quadrant), and necrotic cells (upper left quadrant). Represent results of one of 3 independent experiments. Table resume all results obtained.

Western blot analysis was used to examine the ability of the VHL mutants RCC4-VA to regulate HIF-1α subunits relative to wild-type VHL function (Fig 5). Intensity of the western blot was quantified for each bands density using the LI-COR Aerius scanner (Fig 5B). HIF-1α protein levels were evaluated with immunoblot detection in each of the cell lines. We observed high levels of HIF-1α in the native RCC4 cells and low levels in the wild-type RCC4/VHL cells. Upon treatment with MDN-0066 at 25μM for 24hrs we observed an over expression of HIF-1α in both cell lines but with a higher rate in RCC4-VA cells. The increased sensitivity of the RCC4-VA cell relative to the RCC4-VHL to MDN-0066 treatment correlates with the relative levels of the PKM2 and MCT4 protein levels (Fig 5). These results suggest that it is the diminished ability to suppress the normoxic stabilization of the HIF-1α subunit and the subsequent transcription directed by HIF that accounts for the increased sensitivity to MDN-0066 while PKM2 and MCT4 were over expressed when HIF was inactive [22,23]. MYC is associated with PKM2 and plays a central role in the regulation of gene expression by HIF activation [24] and cell cycle [25]. Perturbation of cell cycle upon treatment with MDN-0066 correlates with a higher increase in MYC expression in RCC4-VHL than in RCC4-VA cells. A similar approach used by Turcotte et al. in 2008 gave rise to a new compound whose mechanism of action involved autophagy [26]. The induction of autophagy was checked by Western blotting for LC3 and Beclin but no effects were observed in either RCC4-VA or RCC4-VHL cell lines. This may indicate that MDN-0066 induces cell death independently of autophagy. On the other hand, our results suggest that MDN-0066 induce apoptosis *in vitro* in VHL-deficient cells as assayed by PARP cleavage (Fig 5) after 48 hrs of treatment. To further investigate the MDN-0066 activation of PARP, cell apoptosis was also assessed by flow cytometry (Fig 6).After 48h treatment with 25 μM MDN-0066, 48± 6% RCC4-VA cells are Annexin V positive while only 5± 2% of similarly treated cells of RCC4-VHL (p < 0.0005). Indeed, 25 μM and 12.5 μM MDN-0066 treatment induce Annexin V staining after 24h and 48h, respectively, in RCC4-VA cell line while no sinificative effect is observed in RCC4 VHL cell line.

Taken together, these results show that MDN-0066 selectively kills RCC cells with a loss of VHL *in vitro* and also significantly reduces tumor growth in VHL-deficient cells.

## Discussion

Natural products with industrial applications can be produced from primary or secondary metabolism of living organisms. Owing to technical improvements in screening programs, as well as separation and isolation techniques, the number of natural compounds discovered exceeds 230,000 [27]. Of all the reported natural products, approximately 20% to 25% show biological activity, approximately 10% have been obtained from microbes, and of these, 45% are produced by actinomycetes, 38% by fungi, and 17% by unicellular bacteria. [27]. The increasing role of microorganisms in the production of antibiotics and other drugs for treatment of serious diseases has been dramatic. Microbial metabolites are among the most important cancer chemotherapeutic agents. Since the discovery of actinomycin in 1940, more than 60% of the current compounds with antineoplasic activity were originally isolated as natural products or are their derivatives [28]. Although there are currently no inhibitors of the VHL mutation in clinical trials, several different natural products [29] have been identified, and strategies are being developed to use them therapeutically.

In this study, we focused our effort on a collection of 1,040 organic extracts obtained from 117 bacterial strains belonging to at least 25 genera of the phyla *Actinobacteria*, *Firmicutes*, *Proteobacteria* and *Bacteroidetes*. These efforts, together with the identification of novel targets and the development of innovative assays that can be easily adapted to HTS technology, constitute the key to the current drug discovery programs. It is in this context that the development of a bioassay based on the differential cell expression of pVHL has a series of remarkable advantages.

In the present report, we have shown that RCC4-VA/RCC4-VHL, a quantitative cell-screening assay suitable for HTS, can be applied to screen libraries of natural products of microbial origin. The assay strategy is based on rescue of the normal function of VHL protein naturally mutated and functional in normal RCC4 cells. In untreated RCC4VA/RCC4VHL cells, the proliferation and survival of both cell lines is equivalent. Compounds that interfere with the VHL-independent cell growth lead to the decrease of RCC4VA proliferation or metabolism whereas no effect is observed in RCC4VHL cell line. Applying this RCC4VA/VHL screening system to a small collection of microbial natural products extracts, we detected several positive extracts with VHL-independent inhibition activity. The results of this work are the first presenting the identification of potential lead compounds from natural products libraries with a lethal interaction in the absence of the von Hippel-Lindau tumor suppressor gene. Although other bioactive lipodepsipeptides, some of them having undergone clinical trials, have been recently identified in other drug discovery programs in search for new antitumour therapies, the discovery of MDN-0066 is the first report of a molecule of this class affecting specifically RCC4VA cells. Bacteria of the genus *Pseudomonas* and *Bacillus* are well known producers of several families of lipodepsipetides such as the viscosins, amphisins, syringomycins, syringopeptins, tolaasins and xantholysins [30]; [31]. These molecules present interesting biological properties related the natural microbial interactions in the environment (antagonism of microbial competitors and facilitation of surface motility, protection against predators, biofilm formation, contribution to virulence of plant pathogens, and triggering of the defense response in plants [32] and it cannot be ruled out that they also might be active in our RCC4VA/VHL screening system. Interestingly, the number of amino acid residues and the size of the cycle place the structure of MDN-0066 within a new structural class never previously reported in Pseudomonas lipodepsipeptides.

We showed that the lipodepsipeptide (MDN-0066) decreases the cell growth of the RCC4VA cells at concentrations that do not affect RCC4VHL cells. IC_50_ for MDN-0066 was 24.4 μM for RCC4-VA and 69.23 μM for RCC4-VHL in this assay. It has been proposed that the resistance of RCC to chemotherapy and radiotherapy is due to increased levels of the nuclear factor kB activity and resistance to apoptosis [33]. In the present study we demonstrate that MDN-0066 can induce apoptosis in cells with loss of VHL has shown by PARP cleavage after treatment of RCC4 VA. This result is confirmed by Annexin V/PI staining showing specific induction of apoptosis in RCC4-VA cells. Previous studies have reported that the metabolic stress observed in human tumors leads cancer cells to acquire resistance to apoptosis and to stimulate autophagy to maintain energy demand and prevent necrosis (Jin et al., 2007). Actives compounds described in the contest of the loss of VHL involved an autophagic process (Turcotte et al., 2008). However MDN-0066 compound has no effect on LC3b and beclin so autophagy is not involved in cell death induction. Thus, MDN-0066 induces cell death in VHL deficient cells with a new type of mechanism of action. Cell cycle arrest in G2/M is observed after MDN-0066 and apoptosis induction is revealed by PARP cleavage detection. Then resistance to apoptosis of RCC after chemotherapy is bypassed by MDN-0066 treatment and absence of autophagy induction prevent a potential cell death escape mechanism. Work is currently underway to further investigate the link between cell cycle arrest and apoptosis induction in the context of RCC4 treatment with MDN-0066. Future studies will explore the therapeutic potential of rapid and/or prolonged inhibition by MDN-0066 in a broad panel of human tumor cell lines. It is particularly important to know whether the inhibition of RCC4 cells by MDN-0066 is a reversible inhibition to limit the toxicity associated with the inhibition of the general VHL/HIF machinery. To summarize, our data demonstrate that RCC4VA/RCC4VHL is a sensitive and robust assay system that allows a rapid and simple screening of extracts containing complex mixtures of natural products. For the first time we demonstrate that VHL mutated RCC4 cell line and a genetically VHL rescued RCC4 cell line can be used in a cell based assay to screen a natural microbial extract collection. As a successful result of this exercise, it was identify a novel inhibitor of VHL/HIF pathway, MDN-0066, that regulates the RCC4 cells metabolism. It represents a new alternative for treatment of kidney cancer inducing cell death of VHL mutated cells by apoptosis without autophagy induction. This MDN-0066 compound is produced by a new *Pseudomonas* species, *P*. *granadensis* sp. nov. [34](. This *Pseudomonas* species newly described by MEDINA foundation scientist represent a novel source of secondary metabolite that can present useful biological activity. Importantly, the approach described in this article can be scaled up to HTS assay format to test large collections of microbial natural product extracts or synthetic compounds.

## Supporting Information

S1 Fig 1H NMR spectrum of MDN-0066. (TIF)

S2 Fig ^13^C NMR spectrum of MDN-0066. (TIF)

S3 Fig MS/MS fragmentation of MDN-0066. (TIF)

S4 Fig Key HMBC (solid line) and NOESY (dashed line) correlations observed for MDN-0066. (TIF)
