# Supplementary figures and images for: Identification of the Lipodepsipeptide MDN-0066, a Novel Inhibitor of VHL/HIF Pathway Produced by a New Pseudomonas Species
Source: PLoS One. 2015 May 27;10(5):e0125221. doi: 10.1371/journal.pone.0125221 (PMC4445906; doi:10.1371/journal.pone.0125221)

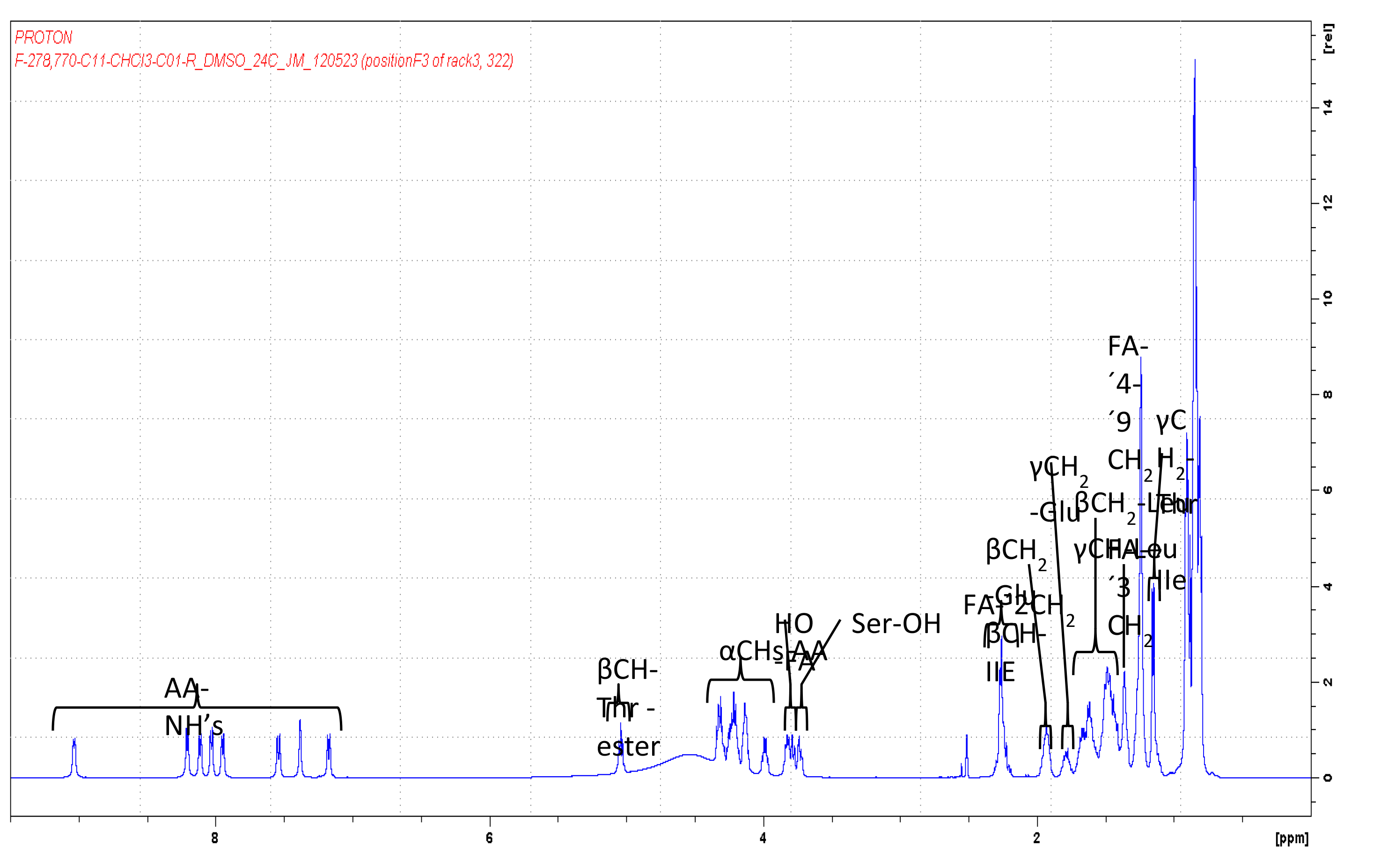

Supplement: S1 Fig — (TIF) [file pone.0125221.s001.tif]

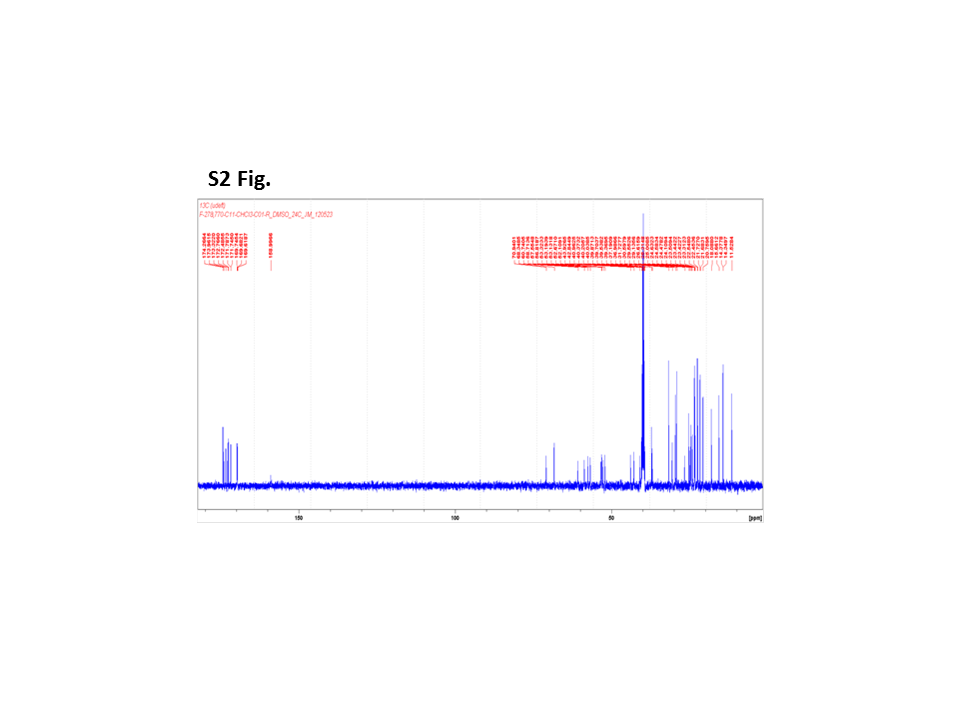

Supplement: S2 Fig — (TIF) [file pone.0125221.s002.tif]

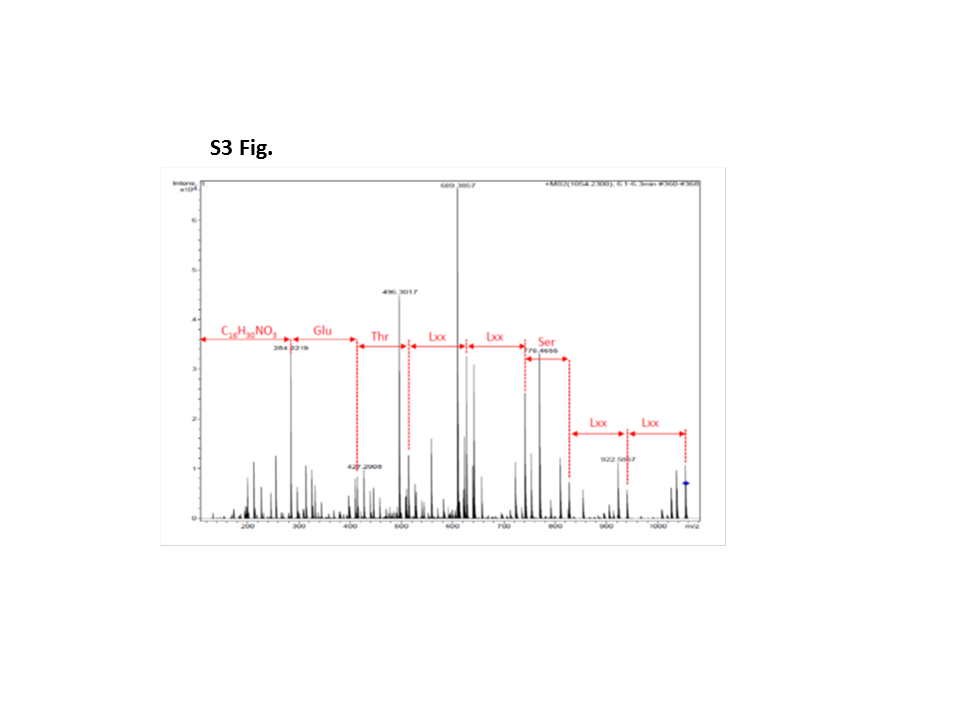

Supplement: S3 Fig — (TIF) [file pone.0125221.s003.tif]

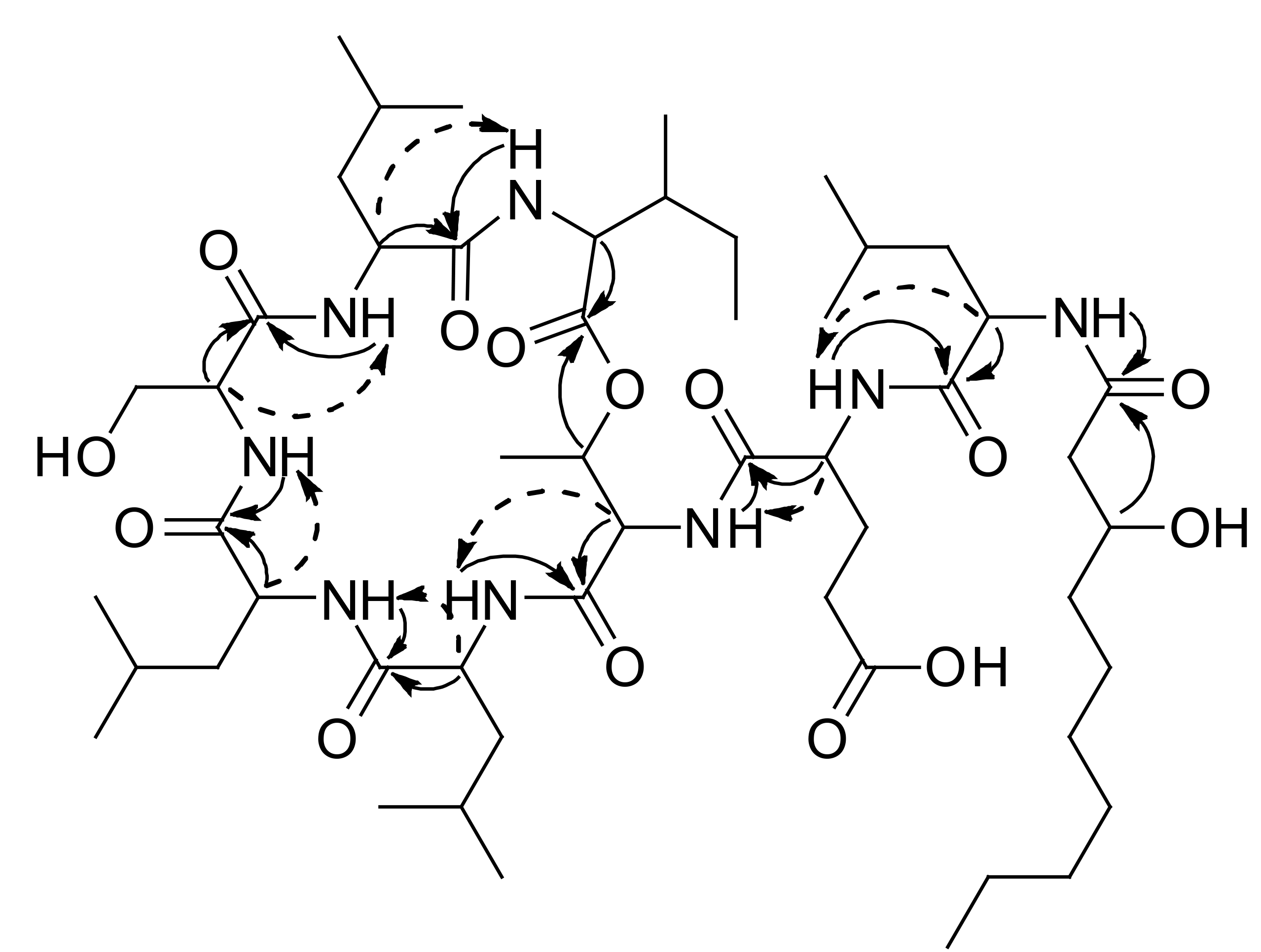

Supplement: S4 Fig — (TIF) [file pone.0125221.s004.tif]
